# Mobile delivery of alcohol treatment: a systematic review of the literature

**DOI:** 10.1186/1940-0640-10-S1-A50

**Published:** 2015-02-20

**Authors:** Andrew Quanbeck

**Affiliations:** 1 The Center for Health Enhancement Systems Studies, University of Wisconsin-Madison, Madison, WI, 53706, USA

## Background

While research supports the effectiveness of continuing care in addiction treatment, the field has historically offered little ongoing support to patients (whether outside of clinic walls during treatment or after patients complete treatment). Mobile technology may make it possible to provide both self-management help and continuing care more widely. This paper seeks to explore the following questions about mobile applications intended for patients recovering from alcohol addiction:

1. What mobile health applications to treat alcoholism exist in the peer-reviewed literature?

2. What features do these applications commonly have?

3. Are the applications integrated with in-person treatment or other aspects of the health-care system?

4. Are the applications theoretically informed and effective?

5. What are the challenges and opportunities facing mobile health for alcoholism?

## Methods

Mobile applications for recovery from alcohol addiction were identified by searching electronic databases of the research literature. The search of electronic literature databases was done using a combination of two keyword sets: (1) “App” or “Apps” or “mobile application*” or “mobile health” or “mhealth” or “text” or “texting” or “text message*” or “messaging” or “smartphone*” or “iphone*” or “Android” or “mobile device*” and (2) “alcohol addiction” or “alcohol abuse” or “alcohol dependence*” or “alcoholic” or “alcoholism” or “alcohol* recovery” or “sobriety” or “sober” or “addiction recovery.” Two researchers independently reviewed the abstracts and selected articles in which mobile applications were mentioned as interventions used to assist recovery from alcohol addiction.

## Results

The researchers reached consensus on 20 articles to include in the review. These articles describe 13 unique mobile systems. They categorized systems in three sets: 1) text-messaging monitoring and reminder systems; 2) text-messaging intervention systems; and 3) comprehensive recovery management systems. The mobile health systems studied cover a broad spectrum of complexity, from relatively simple texting-based monitoring and reminder systems, to comprehensive recovery management support systems. In general, the less complex texting-based systems were designed with minimal theoretical grounding. As more intervention functions were added in more complex systems, communication, behavioral, and social support theories were increasingly brought to bear. Systems that rely primarily on texting have the advantage of being inexpensive, widely available (given the nearly universal penetration of basic mobile phones), and easy to operate for both senders and receivers of text messages. These characteristics make it relatively simple to incorporate texting technology into existing treatment. While texting systems have the advantage of being easy to use, the tradeoff is that they do not appear to be as effective as enhanced systems that use smartphone technology. One explanation for the greater effectiveness of a comprehensive application is that it can provide more modes of treatment and more tools, thereby better addressing individual preferences, leading to better learning and more lasting recovery.

## Conclusions

Mobile health technology presents opportunities for better integration of care, but execution and implementation remain major obstacles. Researchers will be challenged to stay abreast of the rapid pace of technological change as they seek to develop and test mobile health technologies.

## Trial registration

ClinicalTrials.gov NCT01963234

ClinicalTrials.gov NCT01003119

ClinicalTrials.gov NCT01702142

## Grant support

NIAAA 5R01AA017192-05

NIDA 1R34DA036720-01A1

NIDA 5R01DA034279-03

NIDA 5R01DA030431-03

